# Incidence, prevalence, and hybrid approaches to calculating disability-adjusted life years

**DOI:** 10.1186/1478-7954-10-19

**Published:** 2012-09-12

**Authors:** S Andrew Schroeder

**Affiliations:** 1 Department of Philosophy, Claremont McKenna College, 850 Columbia Avenue, Claremont, CA, 91711-6420, USA

**Keywords:** Disability-adjusted life years, Incidence perspective, Prevalence perspective, Burden of disease, Discounting

## Abstract

When disability-adjusted life years are used to measure the burden of disease on a population in a time interval, they can be calculated in several different ways: from an incidence, pure prevalence, or hybrid perspective. I show that these calculation methods are not equivalent and discuss some of the formal difficulties each method faces. I show that if we don’t discount the value of future health, there is a sense in which the choice of calculation method is a mere question of accounting. Such questions can be important, but they don’t raise deep theoretical concerns. If we do discount, however, choice of calculation method can change the relative burden attributed to different conditions over time. I conclude by recommending that studies involving disability-adjusted life years be explicit in noting what calculation method is being employed and in explaining why that calculation method has been chosen.

## Background

When used to measure the burden of disease on a population during a time interval, disability-adjusted life years (DALYs) can be calculated in different ways – or, as it is usually put, from different perspectives. Studies sometimes choose one method of calculation over the others for reasons of convenience (based, say, on what type of data they have access to) [1], and other times don’t even acknowledge the issue, never explicitly saying what calculation method is being used [2-7].^a^ This is, I believe, a serious mistake. Although the point isn’t frequently recognized, the different ways of calculating DALYs yield measurements of different quantities; they are *not* alternative approaches to quantifying the same thing [8]. It is important that a calculation method be chosen with this in mind.

In what follows, I’ll first explain the three main ways in which DALYs can be calculated, from an incidence, pure prevalence, or hybrid perspective, and I’ll show that each method produces a measurement of a different quantity. I’ll then discuss the formal features of each perspective and explain the apparent difficulties that each faces. I’ll show how this issue interacts with another important question in health measurement: whether the value of future health states should be discounted. I’ll show that if we don’t discount, there is a sense in which the perspective issue is a “mere” question of accounting which raises no deep conceptual concerns. On the other hand, if we do discount, then the choice of perspective is crucial. Different choices will result in changes to the relative burden attributed to different diseases or risk factors. I’ll conclude with a few recommendations.

### DALYs and time

All DALYs are calculated by adding YLLs (years of life lost to premature mortality) to YLDs (years of life lived with disability, multiplied by a disability weight [DW] representing the severity of the condition).^b^ This calculation is relatively simple when DALYs are not linked to a time interval but instead are linked to a well-defined population. If we want, for example, to know how many DALYs were lost by the 1890 birth cohort, we can simply count how many years prematurely the members of that cohort died and then determine the duration of disability they experienced, multiplied by severity. Many DALY measurements, however, are linked to time. Instead of looking at how many DALYs have been lost to lung cancer, we often want to know how many DALYs were lost to lung cancer *in 2004*. This kind of measurement is potentially very useful, since it allows us to track changes over time, monitor the impact of policy interventions, and the like. But it also raises a number of problems.^c^

Suppose, then, that we want to determine the number of DALYs lost in 2004 to lung cancer. We know that all DALYs are the sum of YLLs and YLDs. To determine the number of YLLs due to lung cancer in 2004, it seems reasonable to look at all the lung cancer deaths in 2004 and then to count how many years premature each death was. So, if Sally dies from lung cancer in 2004 at age 50, then (assuming a life expectancy of 80 years) that means 30 years of life were lost.^d^ To determine the number of YLDs due to lung cancer in 2004, it seems sensible to look at how many people suffered as a result of lung cancer that year, and then to multiply those person-years by the appropriate DW. So, if Jim experiences mild (DW = 0.2) disability throughout 2004 on account of his lung cancer, then 0.2 DALYs were lost on account of his lung cancer in 2004.

This is, I think, the most intuitive way to think about YLLs and YLDs in 2004, but note that the method – the perspective – adopted for each component was quite different. We calculated YLLs by identifying a collection of *events* (deaths) that took place in our time period and determining the stream of future health losses associated with those events. We calculated YLDs, on the other hand, by looking at the *states experienced* in our time period and adding that time up. Theoretically, it doesn’t seem to make much sense to just add these results together to get the total DALYs lost to lung cancer in 2004, since they are based on very different methods [8,9]. The resulting output wouldn’t measure anything easily describable. It could best be summarized as the future years of life lost to premature mortality due to deaths in 2004 plus the amount of disability experienced in 2004.

What, then, can we do to avoid that kind of chimeric measure? The most common strategy is to change the YLD calculation method to bring it in line with that used to count YLLs. So, instead of looking at the amount of disability experienced in 2004, we instead pick some type of event and look at the future stream of disability connected with events of that type occurring in 2004. We might, for example, look at all disabling events, like we looked at deaths. If I go permanently blind (DW = 0.6) in 2004 at age 30, we could attribute 50 years x 0.6 = 30 DALYs to 2004’s YLD count. Your blindness, however, which struck in 2003, wouldn’t be counted at all; its full impact would have been registered in 2003’s YLD count. This approach is called an *incidence perspective*, and it is the method most commonly used to calculate DALYs. With a few qualifications to be discussed below, I-DALYs (incidence DALYs) for period T measure the stream of lost health connected with events in T.

The second apparent solution to our original puzzle is to take the opposite route and to change the method for calculating YLLs to bring it in line with that used when calculate YLDs [10]. Rather than looking at events that happened in 2004, this would involve counting ill health experienced in 2004. It’s very natural, as we saw, to do this for disability, but it doesn’t make sense in the case of death. No one “experiences” being dead. What we can do, though, is add up the health that would have been experienced in 2004, but for injury, disease, and so forth. That is, we can calculate YLLs for 2004 by counting the number of people who died in the past, whose natural lifespan included 2004. So, the lung cancer death of a 50 year-old in 2000 would count as one YLL for each year from 2001 through 2030, since the deceased would have been alive in each of those years had she not contracted lung cancer and instead lived out her natural lifespan. This approach to calculating DALYs doesn’t have a name, since (to my knowledge) it has never been used. I’ll call it a *pure prevalence perspective* and call DALYs calculated this way PP-DALYs. Roughly, PP-DALYs for period T measure the amount of additional health that would have been experienced in T, but for injury, disease, and so forth.

Finally, the third response to our original puzzle has been to ignore it – when calculate YLLs from an incidence perspective and YLDs from a prevalence perspective and then to simply add the results.^e^ This approach, which is less common than the incidence approach, is usually described in the literature as taking a prevalence perspective, but for reasons which I hope are clear, I will call it a *hybrid perspective* and call the resultant DALYs H-DALYs. As mentioned earlier, H-DALYs don’t seem to measure any nondisjunctively describable quantity.

**Figure 1:**
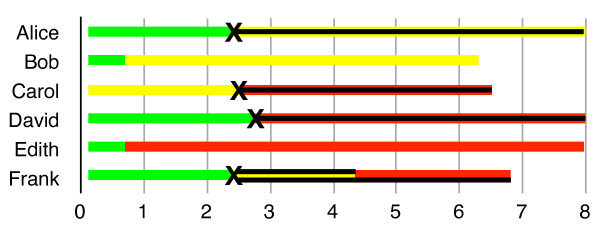
** Incidence DALYs.** To calculate I-DALYs for year #2, look for events – color changes – in year #2, and then add up the future stream of ill health connected to those events. A sequela-based measurement (see below) will count only Frank’s two years of disability; a pathology-based measurement will also count his 2+ years of premature mortality, but will not count Carol’s four years of premature mortality (unless her death was unrelated to her disability).

**Figure 2:**
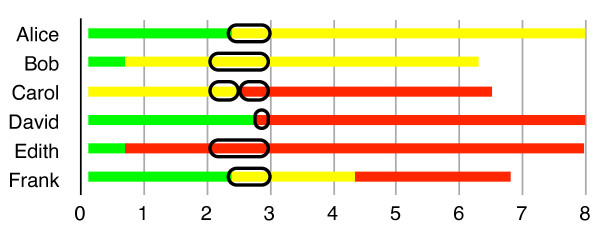
** Pure prevalence DALYs.** To calculate PP-DALYs for year #2, add up all the yellow (disability) and red (premature mortality) that occur in year #2 on the chart.

**Figure 3:**
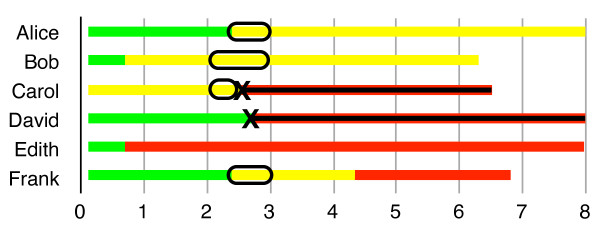
** Hybrid DALYs.** To calculate H-DALYs for year #2, add up all the yellow (disability) that occurs in year #2, and then look for any deaths in year #2, adding up the (future) years of life lost to those deaths.

Visually, all of this is represented in Figures 1, 2 and 3, where green represents time lived in good health, yellow represents time lived in a particular disabled state, and red represents time lost to premature mortality.

### Conceptual choices and problems

Having briefly described the three main approaches to calculating DALYs, I’ll now discuss each in more detail, identifying some of the choices and problems each perspective faces.

#### I-DALYs

DALYs are most commonly calculated from an incidence perspective. This involves selecting some type of event, identifying the events of that type that occur in the target time period, and then adding up the DALYs that are connected to those events. Calculating I-DALYs therefore requires (1) choosing a type of event and then (2) specifying how lost health is connected to events. (These choices are rarely made explicit in the literature.) In order not to undercount lost health, all lost health should be connected to an appropriate event; but in order not to overcount lost health, all lost health should be connected to only one event. Here are four possibilities:

1. Individuation by sequelae and death: “events” are the onset of a sequela or a death; we connect all health lost to a sequela or to death to its onset.^f^

2. Individuation by pathology: “events” are the onset of a pathology; we connect all health lost to a pathology to its onset.

3. Individuation by pathology and death: “events” are the onset of a pathology or a death; we connect all health lost to disability to the onset of its underlying pathology, and we connect all health lost to death to death.

4. Individuation by death: “events” are the death of an individual; we connect all health lost by an individual to her death.^g^

Suppose that Jane begins smoking in 1950. She contracts chronic obstructive pulmonary disease in 1980, which gives her six years of mild disability (DW = 0.2), followed by two years of serious disability (DW = 0.5), followed by death in 1988 at age 60. If we individuate by sequelae and death and assume a life expectancy of 80 years, we’ll assign 6 × 0.2 = 1.2 DALYs to 1980; 2 × 0.5 = 1 DALY to 1986; and 20 × 1 = 20 DALYs to 1988. If we individuate by pathology, we’ll assign all 22.2 DALYs to 1980. If we individuate by pathology and death, we’ll assign 2.2 DALYs to 1980 and 20 DALYs to 1988. If we individuate by death, all 22.2 DALYs would be assigned to 1988.

The first option is the method officially used in the Global Burden of Diseases, Injuries, and Risk Factors (GBD) Study [11]. Many studies claim to be using the third option – while at the same time saying they are following GBD methodology [12].^h^ I mention the second option because it seems more internally consistent than the third and is used for calculating Healthy Life Years (HeaLYs), a measure related to the DALY [13]. The fourth method has never been used and probably never will be. I mention it simply to show that there are many possible ways of calculating I-DALYs. Some less obvious ways may prove to be useful. In the above scenario, for example, we might try to individuate by primary cause or risk factor, assigning the 22.2 DALYs to Jane’s decision to smoke in 1950.

Given, then, the many different possible versions of an incidence approach, it is important to be clear on the method of calculating I-DALYs being used. It is also important to resist the temptation to describe I-DALYs as measuring the amount of ill health lost due to *causes* of a particular type in a time period. This may be true for some methods of event individuation, but it is certainly not true for all of them. (Individuation by pathology comes the closest.) It is therefore more accurate to describe I-DALYs as measuring the amount of lost health *connected with* events in a given time period and then to explicitly define the connection relation.

Finally, note that in no intuitive sense are I-DALYs (of whatever type) a measure of the health of a population at a time or of the burden of disease on a population at a time. Suppose, for example, that last year we were struck by a massive epidemic that caused permanent blindness, paraplegia, and chronic pain in the entire population. If there are no new health problems (incident pathologies, sequelae, or deaths) this year, then zero I-DALYs would be attributed to this year. But if someone asked how healthy we were this year or whether we experienced any burden resulting from disease, we would surely answer that we were not at all healthy and experienced a huge burden, since we’re blind, paralyzed, and in chronic pain. Call this the *polio problem*, since for many years in many countries polio would have registered zero I-DALYs, despite having left many people with serious disabilities.

#### PP-DALYs

Pure prevalence DALYs calculate YLDs by looking at the gap between health actually experienced in some time period and full health, and they calculate YLLs by counting the number of people who died in the past but who would have been alive in the time period had they lived a normal lifespan. PP-DALYs are in a sense simpler than I-DALYs, in that they don’t require defining things like “events” and the connection relation. It is tempting to say that they measure the amount of additional health that would have been experienced in the time period but for pathology, but there are two reasons why that isn’t quite right. First, YLLs are calculated based on a somewhat arbitrary choice of target life expectancy. If we truly had *no* ill health, people would live much longer than 80 years. (In fact, if a fatal condition is considered ill health, then absent ill health no one would ever die.) Second, PP-DALYs are not designed to make this kind of counterfactual assessment. If I become infertile, that infertility will register a (small) number of DALYs each year I experience it. But the health that would have been experienced by the children I would have had, had I been fertile, will not register on a PP-DALY measure.

So, it is more accurate to say that PP-DALYs measure the amount of additional health that would have been experienced in a given time period by past or present members of some population, had they lived to an arbitrary age in good health. As with I-DALYs, this is not an intuitive measure of the health of a population at a time. Elvis Presley died in 1977 at age 42. He, therefore, would still count for one YLL this year. But, intuitively, it does not seem that we as a population are in poorer health now because Elvis died 35 years ago. Call this the *Elvis problem*.

#### H-DALYs

Hybrid DALYs are calculated by adding I-YLLs to P-YLDs. As such, H-DALYs avoid both the polio and Elvis problems. (The polio problem arises due to the use of I-YLDs and the Elvis problem due to the use of P-YLLs.) They also largely sidestep the difficulty facing I-DALYs in choosing “events.” Since only YLLs are calculated from an incidence perspective, it seems natural to select death as the event in question and then to connect all years of life lost to the moment of death. Finally, H-DALYs are a true period measure: all the data needed to calculate H-DALYs can be measured in the time period in question. (I-DALYs require a projection of the future duration of disability, and PP-DALYs require knowledge of deaths that occurred prior to the time period in question.) These are the virtues of H-DALYs, and they are considerable. Unfortunately, there are also serious problems. As we’ve seen, H-DALYs don’t measure any intuitively describable quantity, and they therefore appear to lack theoretical justification.

I believe, however, that H-DALYs may be justifiable as our elusive measure – or, more accurately, index – of population health at a time, a role we saw that neither I- nor PP-DALYs could plausibly fill. Some educational assessment indices for schools include both a component connected to the academic performance of students (e.g., standardized test scores) and a component that reflects the drop-out rate [14].^i^ The thought is that a school has in some sense failed in its mission if its students perform badly on (well-designed) tests or if they drop out. A school’s job is both to increase test scores and to keep students in the system. Similarly, we might think that we, as a population, are doing poorly with respect to health if our members are living with disabilities or if we are allowing our members to “drop out” of the population by dying. The job of our health system is both to decrease disability and to keep people alive. The former component, I suggest, may plausibly be represented with P-YLDs, and the latter with I-YLLs. (I argue for this claim more extensively in an unpublished manuscript, “Measuring Health and the Problem of Changing Populations.”)

### DALYs, accounting, and discounting

Given, then, that we have (at least) three different ways to calculate the DALYs lost for a given time period, which one should we use? There is one sense in which the decision might not seem like a theoretically significant one. As we saw, these issues only arise when we try to assign DALYs to time periods. If we ask, for example, how many DALYs John lost to ill health, there is a simple answer. Similarly, if we ask how many DALYs members of the Boston Red Sox lost to ill health, there is a simple answer. Things only become complicated when we ask how many DALYs members of the Boston Red Sox lost to ill health *in 2010.* Our problem, then, is really just one of accounting. We have a set number of DALYs that have been lost, and we want to assign those DALYs to different time periods. Each of the three methods we’ve looked at provides an internally consistent way of doing that. There aren’t any deep metaphysical issues raised when an accountant decides whether to calculate asset depreciation annually versus allocating the entire loss to the year of sale. Similarly, there is no principled reason we can’t allocate DALYs to time periods in any number of ways, so long as we do so consistently.

That said, there are two reasons why the choice of accounting has at least practical importance. First, the choice of perspective – the accounting method – may make a difference in how certain diseases “look” over time. After a successful vaccine has been developed, certain illnesses will no longer show up in I-DALY measurements but will continue to register in H- and PP-DALY measurements. Certain cyclical diseases with lasting effects may appear only to be sporadic problems from an incidence perspective while appearing to be chronic problems from a pure prevalence or hybrid perspective. H- and PP-DALYs may appear to underestimate the importance of a new, chronic health problem, if most of its effects will occur in the future. Of course, each of these problems can be corrected for by taking a more comprehensive view. (We can correct for the last problem, for example, by also looking at predicted future H- and PP-DALYs.) But in the real world we know that such care won’t always be taken, and so there is potentially some practical importance in the choice of perspective, because of the different “shape” or trajectory it can give to different health problems.

Second, choice of perspective interacts with discounting in a crucial way. The practical matters I mentioned above concern the shape of different DALY measurements. Calculated one way, a disease may show huge periodic “spikes” of DALYs lost; calculated another, it may register a relatively constant drain on the population. But in the end, the total number of DALYs lost over time will be the same, which is why the issues can easily be corrected for by more carefully examining the measurements and taking a broader view. If, however, we discount the value of future health, this need not be the case.

Suppose condition A affects 100 people per year and gives each person exactly one year of serious disability (DW = 0.5). Condition B, which has two incident cases each year, causes 50 years of equally serious disability. If B’s incidence rate has been constant, it will eventually have the same prevalence as A – 100 people will have each condition at any given time. From a hybrid or pure prevalence perspective, both A and B will register 100 × 0.5 = 50 DALYs per year, every year. This will be true whether or not we discount the value of future health, since both H- and PP-DALYs calculate YLDs by looking at the health loss experienced this year.

When we calculate I-DALYs, however, things change. Whether or not we discount, condition A will register 100 cases × 1 year × 0.5 = 50 DALYs each year. If we don’t discount, condition B will register 2 cases × 50 years × 0.5 = 50 DALYs each year. If we do discount, however, we’ll get a much lower number for B, since the heath loss in the future will count for less. (Using a low 3% discount rate, B will register 25.9 I-DALYs each year.) This means that if we don’t discount, then H-, PP-, and I-DALYs will all measure A and B as equally serious health problems – 50 DALYs per year, every year. If we do discount, H- and PP- DALYs will still count A and B equally, but I-DALYs will register A as a much more serious health problem than B (50 vs. 25.9 DALYs). (See Table 1). So, if we discount the value of future health, then the choice of perspective becomes crucial – it can have a huge effect on the relative burden assigned to different health problems, and this difference will not wash out over time. The choice of perspective here no longer seems like mere accounting.^j^

**Table 1:** The impact of discounting and perspective choice on the measured burden for two sample conditions

**Condition**	**Incidence**	**Duration**	**Prevalence**	**H- or PP-DALYs (w/or w/out discounting)**	**I-DALYs (w/out discounting)**	**I-DALYs (3% discount)**
A	100	1	100	50	50	50
B	2	50	100	50	50	25.9

## Conclusions

I’ve explained the many different ways to calculate DALYs – from a pure prevalence, hybrid, or incidence perspective (the last of which has several possible forms) – and I’ve explained some of the conceptual choices and problems that arise for each method. Returning to the question that opened the last section: which is the right one to use? Which perspective should we take when measuring health? I think the answer, unsurprisingly, is that since each measures a different quantity, each is useful in different situations. If we are interested, for example, in estimating the impact of lung cancer on this year’s economic productivity, PP-DALYs are likely a useful data point. H-DALYs may be a good choice when evaluating the performance of a health care system, and I-DALYs might be the best option if we’re deciding how much money to allocate to different vaccine programs. The important point to keep in mind is that PP-, H-, and I-DALYs measure different things. That is, it is not the case that we have a single quantity to measure, *population health in 2004* or the burden of disease in 2004, for which we have three candidate calculation methods. Rather, we have three candidate quantities that we could measure. When undertaking a measurement of health, the first question needs to be: *what, exactly, do I want to measure?* A careful answer to this question will much of the time suggest the proper calculation method.

Two further recommendations are in order. First, those measuring health need to clearly describe their calculation method. As has been noted, many studies do not explain (much less justify) what calculation method has been chosen, and those that do frequently relegate the explanation to a footnote or appendix. For studies adopting an incidence approach, it is very rare to find an explicit mention of what “events” are being counted and of how lost health is tied to those events. These choices matter. It is important for them to be made clear so that results can be properly interpreted and to ensure that only properly comparable results are compared.

Second, I think it is important to speak more clearly about DALYs and what they do and don’t measure. As we noted above, neither I-DALYs nor PP-DALYs are plausible candidates for measuring population health at a time – recall the polio and Elvis problems – and so we shouldn’t describe them as such. Rather, we should explicitly describe I-DALYs for time period T as measuring the health loss connected with events (of whatever type) occurring in T – or, somewhat more loosely, as measuring the total health loss tied to new health problems in T. And we should describe PP-DALYs for T as, loosely, measuring the amount of additional health that would have been experienced in T but for injury, disease, and so forth. What about H-DALYs? I suggested that they can be described as an index representing overall population health for the period. This clarity of description has a cost, of course. It is much easier, and more likely to resonate with the public and with policymakers, to simply describe DALYs as measuring population health or the burden of disease for some period of time. But if that simplicity comes at the price of misunderstanding, I question whether it is worth it.

## Endnotes

^a^Though none of these articles explicitly indicate which calculation method is being used, in some (but not all) cases it can be inferred from the paper.

^b^Disability weights raise a number of important issues which I will set aside here. In particular, there is a question about what a disability weight is supposed to represent – a quantity of health, the value of health states, the well-being associated with health states, etc. The resolution of this issue will affect much of what I say below, about what each type of DALY seeks to measure, in ways which should be straightforward.

^c^For the remainder of the paper, whenever I speak of DALYs, assume that I mean DALYs-for-time-period-T. In particular, many of the recommendations I make at the end of the paper do not necessarily apply to DALY measurements that are not restricted to a time period.

^d^The choice of life expectancy is another important, difficult, and potentially problematic issue. But I will ignore it here.

^e^There is, logically, a fourth possibility: adding P-YLLs to I-YLDs. I know of no one who has even mentioned the possibility, though, and I can’t think of any context in which it would be useful.

^f^There is a sense in which we could simply call this “individuation by sequelae,” where we consider death to be a sequela. Since it is standard practice, however, to reserve “sequela” for nonfatal conditions, I have chosen the slightly longer formulation here.

^g^In each of these cases, further work needs to be done properly operationalize it. What distinguishes one sequela from another? (Is moderate back pain a different sequela than severe back pain?) What counts as the onset of a pathology? (Is it the initially asymptomatic cancerous mutation or the appearance of symptoms?) What counts as health lost to a pathology? (If I accidentally drink from your glass because I’m blind and as a result catch a cold, should the resultant health loss be attributed to my blindness?) These are important questions, but I’ll set them aside.

^h^Even the GBD Study itself makes this mistake. The 2004 update describes YLD as “years lost due to disability…for incident cases of the disease or injury” [12]. Theo Vos (personal communication) has explained to me that in the GBD Study, as well as in some national burden of disease studies, an inconsistent mix of individuation by sequelae and death and individuation by pathology and death has been used. For example, in past GBD studies foot amputation from diabetes was treated as a separate sequela, with YLD assigned to the time of amputation. But YLD owing to different sequelae arising from cancer were assigned to the original onset of cancer.

^i^California’s Academic Performance Index (API) originally included only components relating to student achievement, creating perverse incentives for schools to encourage poor performers to drop out or transfer. Recent legislation has mandated that the API to be revised to include a component reflecting a school’s retention rate [14].

^j^Dan Brock (personal communication) has raised the question of whether these observations are relevant to the much-debated issue of whether or not we should discount the value of future health. There certainly is a sense in which the perspective issue would be simpler if we did not discount. Then, the choice of perspective would really be one of accounting – it wouldn’t affect the relative burden over time attributed to different conditions. So, dispensing with discounting would be a way of making the perspective issue much less pressing, and that might be thought to count in favor of doing so. I suspect, however, that this consideration by itself wouldn’t sway many advocates of discounting, since it is already well known that discounting will shift the relative burden attributed to different conditions. (The cost effectiveness of vaccination programs and programs to alleviate the effects of climate change, for example, are notoriously sensitive to the choice of discount rate.) I think the relevance of the perspective issue to the question of discounting, though, is worthy of further investigation.

## Abbreviations

DALY: Disability-adjusted life year; DW: Disability weight; GBD: Global Burden of Disease; H-DALY: Disability-adjusted life years calculated from a hybrid perspective; I-DALY: Disability-adjusted life years calculated from an incidence perspective; PP-DALY: Disability-adjusted life years calculated from a pure prevalence perspective; YLD: Years of life lived with disability (multiplied by disability weight); YLL: Years of life lost.

## Competing interests

The author declares that they have no competing interests.

## Author’s information

The author is a philosopher who works on issues in ethics and the philosophy of science connected to health measurement.
